# Metabolic Contrasts: Fatty Acid Oxidation and Ketone Bodies in Healthy Brains vs. Glioblastoma Multiforme

**DOI:** 10.3390/ijms25105482

**Published:** 2024-05-17

**Authors:** Corina Tamas, Flaviu Tamas, Attila Kovecsi, Alina Cehan, Adrian Balasa

**Affiliations:** 1 Doctoral School of Medicine and Pharmacy, “George Emil Palade” University of Medicine, Pharmacy, Science and Technology, 540142 Targu Mures, Romania; corina.hurghis@umfst.ro; 2 Department of Neurosurgery, Emergency Clinical County Hospital, 540136 Targu Mures, Romania; adrian.balasa@umfst.ro; 3 Department of Neurosurgery, “George Emil Palade” University of Medicine, Pharmacy, Science and Technology, 540142 Targu Mures, Romania; 4 Department of Morphopathology, “George Emil Palade” University of Medicine, Pharmacy, Science and Technology, 540142 Targu Mures, Romania; attila.kovecsi@umfst.ro; 5 Department of Morphopathology, Emergency Clinical County Hospital, 540136 Targu Mures, Romania; 6 Department of Plastic, Esthetics and Reconstructive Surgery, Emergency Clinical County Hospital, 540136 Targu Mures, Romania; alina.hurghis@yahoo.com

**Keywords:** glioblastoma, fatty acids, ketone bodies, lipid metabolism, β-oxidation, carnitine

## Abstract

The metabolism of glucose and lipids plays a crucial role in the normal homeostasis of the body. Although glucose is the main energy substrate, in its absence, lipid metabolism becomes the primary source of energy. The main means of fatty acid oxidation (FAO) takes place in the mitochondrial matrix through β-oxidation. Glioblastoma (GBM) is the most common form of primary malignant brain tumor (45.6%), with an incidence of 3.1 per 100,000. The metabolic changes found in GBM cells and in the surrounding microenvironment are associated with proliferation, migration, and resistance to treatment. Tumor cells show a remodeling of metabolism with the use of glycolysis at the expense of oxidative phosphorylation (OXPHOS), known as the Warburg effect. Specialized fatty acids (FAs) transporters such as FAT, FABP, or FATP from the tumor microenvironment are overexpressed in GBM and contribute to the absorption and storage of an increased amount of lipids that will provide sufficient energy used for tumor growth and invasion. This review provides an overview of the key enzymes, transporters, and main regulatory pathways of FAs and ketone bodies (KBs) in normal versus GBM cells, highlighting the need to develop new therapeutic strategies to improve treatment efficacy in patients with GBM.

## 1. Introduction

Glucose is an essential nutrient, dependent on blood flow for a constant supply and normal homeostasis of the body. It is the substrate that enters tissue cells and is transformed into adenosine triphosphate (ATP). ATP occupies a key position both in the metabolism of normal cells and in the tumor microenvironment. To maintain the cellular function of the brain, an adequate and continuous supply of energy is necessary, because glycogen is stored in a limited amount at this level. During periods of limited glucose availability, tissues use fat-derived ketone bodies (KBs) as alternative fuel sources [1,2,3,4].

Ketones, the result of lipid metabolism, provide 5% to 20% of the total energy expended by the human body. In the liver, fatty acids (FAs) are transformed into KBs, which then circulate through the bloodstream to various organs, including the brain. When the insulin level is low and there is a high concentration of FAs in the blood, they are converted into KBs providing an alternative source of energy for the body and especially for the brain. Acyl-Coenzyme A (CoA) oxidation takes place in the mitochondria of organs with high energy requirements and generates KBs (β-hydroxybutyrate-BHB and acetoacetate-AcAc). BHB represents approximately 70% of circulating ketones, crossing the blood–brain barrier (BBB) and serving as fuel at this level when needed. At the same time, BHB also acts as a signaling molecule in many cellular functions, including the epigenetic regulation of gene transcription. The carnitine shuttle system facilitates the transport of fatty acyl-CoA in the mitochondria, being a particularly vital mechanism when the blood glucose level is low, ensuring a sustained energy supply of the body [5,6,7,8,9,10,11,12].

Growing evidence supports the link between disturbed lipid metabolism and cancer. Metabolic irregularities favor tumor proliferation. Identifying gene expression changes of enzymes involved in metabolic pathways could widen the range of cancer biomarkers and therapeutic approaches [13,14,15].

Mitochondria have recently been recognized as the “engine of cell death” due to their essential involvement in programmed cell death. Besides the essential role they have in the production of ATP, the generation of reactive oxygen species (ROS), and the facilitation of cell death pathways, mitochondria are involved in various pathological conditions, such as cancer, neurodegenerative diseases, obesity, or diabetes. Mitochondria are the point of convergence between the metabolism of glucose, glutamine, and lipids. The main function is to support the tricarboxylic acid cycle (TCA) and aerobic respiration through oxidative phosphorylation (OXPHOS) which generates ATP through the mitochondrial respiratory chain, thus satisfying the need for energy for cell survival [16,17,18].

Tumor cells undergo metabolic changes that cause these cells to use more glucose than normal cells, transforming glucose into lactate through aerobic glycolysis instead of metabolizing glucose through OXPHOS to produce ATP, a phenomenon known as the Warburg effect. This phenotype implies a high level of fermentation even when oxygen is abundant [19,20,21,22].

Gliomas are the most common primary brain tumors. The World Health Organization (WHO) has classified gliomas into four grades (I–IV), with higher grades indicating increased dedifferentiation and malignancy. Glioblastoma multiforme (GBM) constitutes a significant part (54.7%) and stands out as the most aggressive form, and is associated with high invasiveness, rapid growth, rapid spread in brain tissue, high recurrence rate, resistance to apoptosis, and an unfavorable poor prognosis. Although current treatment involves surgery, radiation therapy, and chemotherapy, the prognosis remains poor, with a median survival of approximately 12 to 18 months and a five-year survival rate of approximately 4.7% [23,24,25,26,27].

This treatment approach does not lead to long-term disease remission for the patients, in part due to the molecular heterogeneity and plasticity of GBM cells, seen present not only between different tumors but also within the same tumor. The metabolic alterations of GBM are attributed to mutations in tumor suppressor genes and oncogenes together with the impact of the surrounding microenvironment, so the interest in studying GBM metabolism as well as the metabolism of the surrounding microenvironment has increased in the last decade. Therefore, it is crucial to develop new therapeutic strategies for GBM patients, which are able to enhance the efficacy of existing treatment modalities while preserving the normal integrity of brain tissue. From this point of view, the knowledge about tumor metabolism and especially about lipid metabolism requires deepening [6,28,29,30,31,32].

In this review, we aim to provide an overview of the current knowledge of fatty acid oxidation (FAO) and its associated pathways in the normal brain versus changes in GBM.

## 2. Fatty Acid Homeostasis

**Figure 1:**
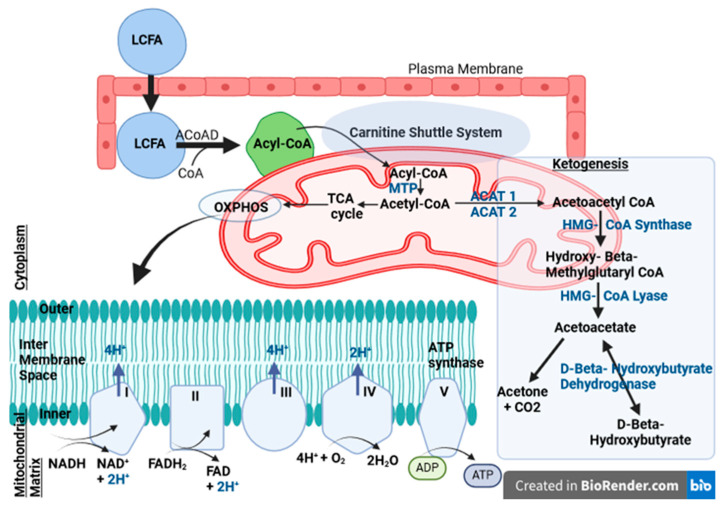
β-oxidation of FAs, Ketogenesis, and OXPHOS. Upon entering the cell, LCFA undergoes activation by binding with CoA. The initial reaction is catalyzed by ACoAD. This transformed molecule, now an acyl-CoA, is transported across the mitochondrial membrane through the carnitine shuttle system. The long-chain acyl-CoA produced is subsequently oxidized to generate acetyl-CoA, a process facilitated by MTP. Created in BioRender.

FAO is a key catabolic pathway that occurs in the mitochondrial matrix for energy production in mammals (see Figure 1). This aerobic process begins with the activation of FAs by linking the thioester with CoA. Mitochondria use three primary enzymatic pathways for ATP generation, namely: TCA, OXPHOS, and FAO (Figure 1). The obvious interaction between these metabolic pathways leads to the maintenance of normal homeostasis of the body [33,34,35,36].

### 2.1. β-oxidation of Fatty Acids

**Figure 2:**
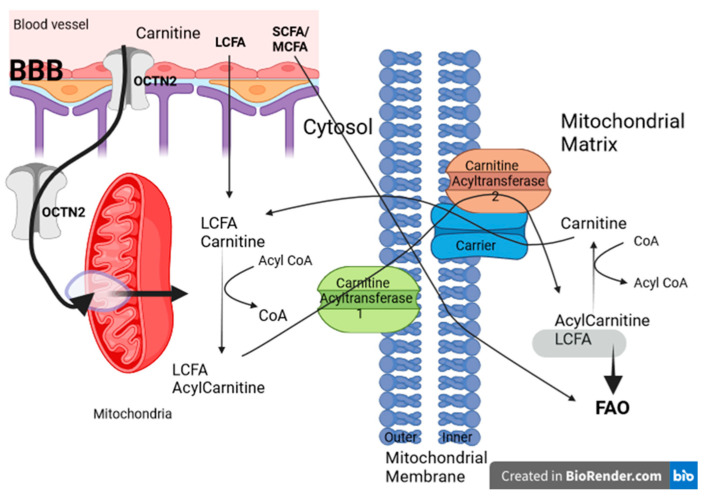
The Carnitine shuttle system for transporting LCFA acyl CoA into the mitochondrial matrix. LCFA is transported across the outer mitochondrial membrane with the assistance of carnitine. Facilitated by the enzyme CPT I located on the outer mitochondrial membrane, FAs bind to the hydroxyl group of carnitine, forming fatty acyl-carnitine. This complex is then transported into the mitochondrial matrix in exchange for carnitine via a carrier protein. Within the matrix, CPT II situated on the inner mitochondrial membrane facilitates the transfer of the acyl group from fatty acyl-carnitine to CoA, resulting in the formation of fatty acyl-CoA and free carnitine. The liberated carnitine is then transported back to the intermembrane space via a carrier protein, where it can be utilized for subsequent cycles. The fatty acyl-CoA within the mitochondrial matrix is now primed for β-oxidation by the enzymes present, ultimately yielding acetyl-CoA. Acetyl-CoA subsequently enters the Krebs cycle, contributing to energy production. OCTN2 is a carnitine-specific transporter found in the brain. Created in BioRender.

β-oxidation of lipids in the mitochondrial matrix involves the removal of a hydroxyl group from FAs, with the formation of highly polar thioesters known as acyl-CoA molecules. Long-chain fatty acids (LCFAs) face a barrier to the free crossing of mitochondrial membranes. To overcome this, LCFAs require transport as carnitine derivatives, called acylcarnitines or esterified carnitines, which facilitate their transport across the mitochondrial membrane (see Figure 2). The LCFA acyl-CoA produced is subsequently oxidized to generate acetyl-CoA, a process facilitated by the mitochondrial trifunctional protein (MTP). Short- and medium-chain fatty acids (SCFA and MCFA) passively diffuse across the mitochondrial membrane and are converted to acyl-CoA esters in the mitochondrial matrix [12,33,35,37].

Inside the mitochondria, the β-oxidation of FAs is carried out through a series of four catalysis reactions. Acyl-CoA dehydrogenase (ACoAD) catalyzes the first step by generating reducing equivalents that are transferred to the electron transfer flavoprotein, which serves as a shuttle between AcoAD and the respiratory chain. Enoyl-CoA hydrase catalyzes the second step, the hydration of 2-trans-enoyl thioesters in 3-l-hydroxyacyl-coA derivatives. In the third stage, the catalysis by Hydroxyacyl-CoA dehydrogenase takes place, obtaining the oxidation of 3-l-hydroxyacyl-coA esters into 3-ketoacyl-coA species. In the last step, under the action of ketoacyl-CoA thiolase, the thiolytic cleavage of the 3-ketoacyl-coA chain by the thiol group of a second molecule of coenzyme A occurs. This sequential process cleaves two carbon atoms from the acyl chain during each iteration, producing, finally, acetyl-CoA molecules. MTP located on the inner side of the mitochondrial membrane and composed of α and β subunits, catalyzes the last three reactions in the β-oxidation of LCFA and MCFA acyl-coA esters. The α subunit catalyzes two consecutive steps in LCFA oxidation, namely, enoyl-CoA hydration and 3-hydroxyacyl-CoA dehydrogenation, while the β subunit has long-chain 3-ketoacyl-CoA thiolase activity. In some types of cancer, it has been shown that nonsteroidal anti-inflammatory drugs can inhibit the multiplication of tumor cells by binding and suppressing the α subunit of MTP. Therefore, it is hypothesized that the selective inhibition of the α subunit of MTP would be associated with the inhibition of tumor growth and therefore could offer new cancer therapies [11,33,38,39,40,41].

### 2.2. Free Fatty Acids and Lipid Droplets

FAs serve as constituents of cell membrane phospholipids and as a fuel source for OXPHOS. Free fatty acids (FFAs) can enter the brain where they can be used as an energy source participating in various physiological processes such as cell transport, cell signaling and transduction, synaptic transmission, protein stabilization, and others, but they can also initiate various harmful activities inside brain cells. Brain FFAs also influence cell growth, development, and survival as well as the inflammatory response at the brain level by regulating the phosphoinositide 3-kinase (PI3K) pathways, the peroxisome proliferator-activated receptor, protein-coupled receptors, protein kinase C, or nuclear factor kappa-amplifying light chain of activated B cells. Active neuronal cells struggle to use FFAs for ATP production, but if neuronal mechanisms are overwhelmed, FFAs can become toxic to neurons. To prevent neuronal damage, FFAs will be stored in intracellular lipid droplets (LDs). Excess FFAs are transported by apolipoproteins in astrocytes, which are abundant in LDs and less vulnerable to the damaging activity of ROS compared to neurons. Astrocytes are considered the primary sites for FFA storage and metabolism in the brain. LDs serve as energy reservoirs, transporting FAs to the mitochondria during nutrient deprivation, where they are used as an alternative energy source. Therefore, to protect neurons from FFA-associated lipotoxicity and to meet energy demands in specific situations, FA storage and oxidation processes appear to rely on a tightly interconnected metabolic relationship between neurons and astrocytes [42,43,44,45].

LDs contain neutral lipids, predominantly triacylglycerols, cholesterol esters, and sterol esters, which are found in most cell types, being synthesized at the level of the endoplasmic reticulum membrane. The process of composition and storage of neutral lipids in LDs serves as a protective mechanism against lipotoxicity induced by excessive lipid accumulation. Each step in the formation of neutral LDs is facilitated by a specific enzyme, with diglyceride acyltransferase I and II (DGAT1, DGAT2) playing an essential role in catalyzing the final step of the triglyceride formation pathways. The two enzymes are found mainly in the endoplasmic reticulum where they catalyze the conversion of diacylglycerol to triacylglycerol, but also in LDs during their growth. It has been shown that the suppression of DGATs promotes axon regeneration because FAs are redirected towards the synthesis of phospholipids at the expense of the synthesis of neutral lipids. DGAT1 also prevents mitochondrial dysfunction and lipotoxicity, which can occur in the case of brain pathology [42,43,44,46].

Physiologically, the formation of LDs is linked to the nutritional state of the cells. In the case of a high availability of exogenous lipids, LDs will form. Also, in case of a lack of an exogenous intake rich in fats, the cells change their energy source from glucose to FAs, so LDs will be formed. But besides these physiological states, LDs are also formed in case of cellular stress such as inflammation, hypoxia, endoplasmic reticulum stress, or mitochondrial dysfunction. The formation of LDs in certain pathological processes presented distinct functional phenotypes, depending on the context or the cell type involved [42,46,47,48].

### 2.3. Carnitine Shuttle System in Normal Mitochondria

The carnitine shuttle represents a specialized mechanism that facilitates the transfer of LCFA through the inner mitochondrial membrane to the mitochondrial matrix for β-oxidation and energy production. FAs come from three main sources: exogenous FAs that enter cells from the bloodstream or intestinal lumen; FAs synthesized endogenously from acetyl-CoA by de novo synthesis; and FAs released inside the cell by hydrolysis of acylated proteins, phospholipids, and triglycerides [5,12,38,49,50].

Regardless of their origin (either exogenous, synthesized de novo, or by intracellular hydrolysis), intracellular FAs undergo thioesterification to CoA. Acyl-CoA synthases (ACoASs) catalyze this process, leading to the formation of acyl-CoA products, the activated form of intracellular FAs [51,52,53].

ACoASs typically associated with proteins and membranes are directed to or away from specific metabolic pathways depending on the cellular energy state. Their intracellular flow and destination are regulated by various proteins, including FA-binding proteins, sterol carrier protein 2, and acyl-CoA-binding domain proteins, which guide them to energy storage or generation processes [50,54,55].

When cells require energy, acyl-CoAs can be transported into mitochondria and peroxisomes, where they collaborate to maintain lipid homeostasis. Substrate transport, substrate specificity, end products, and energy production show variation between the mitochondrial and peroxisomal β-oxidation pathways [56,57].

The impermeability of mitochondrial membranes to acyl-CoA requires the conjugation of FAs to carnitine for their entry into mitochondria. The first component of the carnitine shuttle is Carnitine Acyltransferase I (CPT I) located in the inner part of the outer mitochondrial membrane with the role of converting acyl-CoAs into acylcarnitines and at the same time, a role in the rate-limiting stage of FAO. The second component of the carnitine shuttle, the inner mitochondrial membrane protein, is the Carnitine Acylcarnitine Translocase (carrier) which facilitates the exchange of acylcarnitines and carnitine between the outer and inner mitochondrial membranes. On the matrix side of the inner mitochondrial membrane, the third important component of the shuttle is located, namely, Carnitine Acyltransferase II (CPT II), which is responsible for converting acylcarnitine back into acyl-CoAs to allow subsequent oxidation processes. The carnitine released in this process is translocated back into the cytosol by the same carrier of the inner mitochondrial membrane through an acyl-carnitine/carnitine antiport reaction (see Figure 2) [12,38,58].

Carnitine transporters are encoded by two solute carrier (SLC) gene families, namely, SLC6 (SLC6A14) and several members of SLC22 (SLC22A1, A2, A3, A4, A5, A7, A16). OCTN2/SLC22A5 stands out as a high-affinity carnitine transporter. Carnitine Acylcarnitine Translocase is the A20 member of the SLC25 protein family, proteins that are mainly located in the inner mitochondrial membrane. In one-third of these, their transported substrates are unknown. The expression of Carnitine Acylcarnitine Translocase is found in tissues with high energy expenditure such as heart muscles or skeletal muscles, and, of course, in the liver when glycogen reserves have been exhausted, but also in the brain, even if at a lower rate [32,59,60,61].

The balance of carnitine homeostasis is maintained by a harmonious interplay of dietary absorption, endogenous biosynthesis, and efficient renal reabsorption. The brain does not directly use FAs for oxidative metabolism. Instead, it relies on KBs derived from acetyl-CoA and acetoacetyl-CoA, which are generated via FAO that occurs mainly in the liver [12,38,53,62].

### 2.4. Ketogenesis

Ketogenesis, contrasting with ketolysis, is a biochemical process occurring in the mitochondria, where acetyl-CoA is utilized to produce KBs (see Figure 1) [1,63,64].

Acetyl-CoA acetyltransferase (ACAT) consisting of two metabolic enzymes, one cytosolic (ACAT2) and one mitochondrial (ACAT1), facilitates the reversible conversion of two molecules of acetyl-CoA to acetoacetyl-CoA (Figure 1). HMG-CoA synthetase, which is regulated by succinylation, desuccinylation for short-term control, and transcriptional regulation for long-term control, catalyzes the chemical reaction that leads to the formation of hydroxy-beta-methylglutaryl-CoA (HMG-CoA). Factors such as nutrition and hormones influence these mechanisms, explaining the prevalence of ketogenesis in conditions such as diabetes, starvation, or intense lipolysis [13,64,65,66,67,68].

After this, HMG-CoA lyase catalyzes the conversion of HMG-CoA to AcAc. AcAc can either undergo non-enzymatic decarboxylation in acetone or be converted to BHB by the action of BHB dehydrogenase (BHD). BHD shows the highest activity in the liver, followed by the kidney, heart, brain, and skeletal muscle. This variation in activity can be attributed to its role in catalyzing both the final stage of ketogenesis, predominantly in hepatocytes, and the initial stage of ketone oxidation in extrahepatic tissues [69,70,71].

AcAc and BHB serve as the body’s two primary KBs for energy production. In extrahepatic tissues, including the brain, BHB is converted to AcAc by BHD, and AcAc is converted to acetyl-CoA by beta-ketoacyl-CoA transferase. Acetyl-CoA enters the citric acid cycle, leading to the production of ATP molecules through OXPHOS. However, acetone does not undergo conversion back to acetyl-CoA and is either excreted in the urine or exhaled (Figure 1) [72,73,74,75,76,77].

## 3. Ketone Body Metabolism

Energy is essential for normal brain function and constitutes approximately 20% of the body’s total energy expenditure at rest, despite the brain comprising only about 2% of the total body weight [8,78]. Under typical physiological circumstances, the brain depends primarily on glucose for the production of ATP from the oxidation of glucose. When the availability of glucose is limited, KBs become the vital substrate for the brain, being able to provide up to 60% of the brain’s energy needs. Together with lactate, it serves as the primary alternative fuel for the brain. Both KDs and lactate can cross the BBB via monocarboxylate transporters (MCTs) present in endothelial cells and astroglia [8,79,80,81].

The use of ketones by the brain appears to be regulated mainly by their concentration in the bloodstream. Plasma ketone levels contribute to less than 5% of brain metabolism. Previous research suggests that, unlike healthy tissues, cancer cells are inefficient in using KBs for energy production. Several dietary approaches, such as ketogenic diets, ketogenic MCFA intake, or exogenous ketone supplementation, can produce substantial changes in normal brain metabolism but not in cancer metabolism [8,82].

Currently, very little is known about the regulation and use of KBs at the biochemical level.

### 3.1. Ketone Bodies Enter the Brain through MCTs

KB transport across the BBB is carrier-dependent and does not increase with neuronal activity, unlike glucose transport. Instead, it is correlated with circulating concentrations. MCTs are the known transporters exclusive to KBs and are widely distributed throughout the brain. MCTs are a group of 14 transmembrane proteins encoded by the SLC16A gene family, and eight of them are expressed in the brain. These conveyors can facilitate the movement of a wide range of substrates. MCT1, MCT2, MCT3, and MCT4 are the MCTs responsible for KB (AcAc and BHB) movement across the plasma membrane (see Table 1 for molecular aspects of the MCT family) [8,82,83,84].

**Figure 3:**
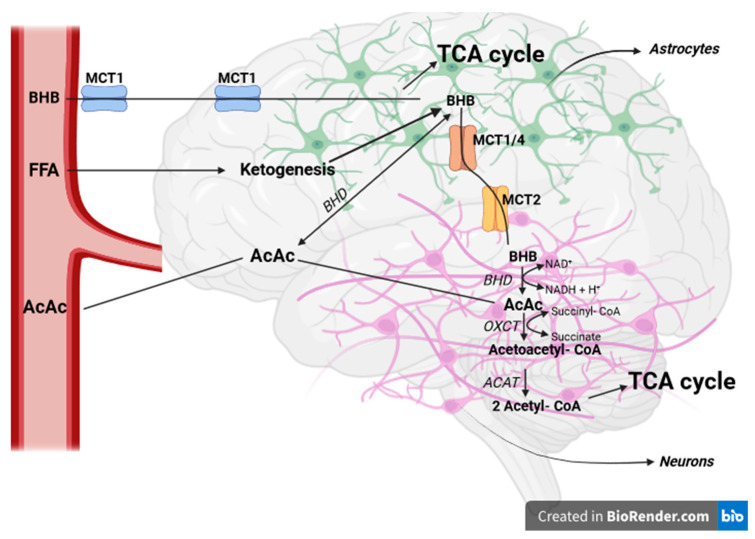
The transport of KBs and FFAs in the brain and the role of MCT. KBs are transported across the BBB via specialized carriers—MCTs, particularly MCT1, MCT2, MCT3, and MCT4. Neurons primarily express MCT2, while astrocytes mainly express MCT4. Within brain cells, KBs are converted back into acetyl-CoA to fuel ATP production in the mitochondria, without the need for ATP input. Created in BioRender.

Astrocytes express MCT4, which, similar to MCT1, has a relatively low affinity for BHB. In contrast, neurons predominantly express the MCT2 isoform, characterized by a high affinity for BHB. The effect of KBs on neurons could still be mediated by neuronal uptake. MCT2 expression in neurons is co-localized in mitochondria-rich postsynaptic density structures, suggesting its potential role in synaptic transmission. This implies that both neurons and, to some extent, astrocytes have the capacity to take up KBs (Figure 3) [8,90,91,92].

### 3.2. The Catabolism of KBs in Glial and Neuronal Cells

After being transported into the brain, BHB and AcAc are converted back into acetyl-CoA, which then enters the TCA cycle for ATP generation (see Figure 3). This conversion takes place within the mitochondria, where BHB is transformed into AcAc by the reversible action of BHD using NAD+, leading to the formation of NADH. AcAc is then broken down to acetoacetyl-CoA by 3-oxoacid CoA-transferase 1 (OXCT1). mRNA levels of OXCT1 are detectable in all human tissues except the liver; hence, the liver cannot utilize KBs as an energy substrate. Acetoacetyl-CoA is subsequently converted back into two acetyl-CoAs, ready to enter the TCA cycle, by the reversible action of ACAT (the first enzyme of ketogenesis). Unlike glucose, this conversion of BHB and AcAc into an oxidizable form does not necessitate ATP. In the developing rodent brain, cultured neurons, astrocytes, and oligodendrocytes all demonstrated the ability to utilize KBs for oxidative metabolism at rates considerably higher than those for glucose. However, neurons and oligodendrocytes appeared to be more efficient at oxidizing ketones than astrocytes [8,93,94].

## 4. Dysregulation of Fatty Acid Metabolism in GBM

Mitochondrial dysfunction in cancer leads to increased OXPHOS activity in these cells. Research has shown that in glioblastoma, mitochondria play a key role in inducing resistance to Temozolomide. Therefore, targeting the treatment of mitochondria could be effective in the treatment of cancer due to the dependence of tumor cells on mitochondria [34,75,76,77].

**Figure 4:**
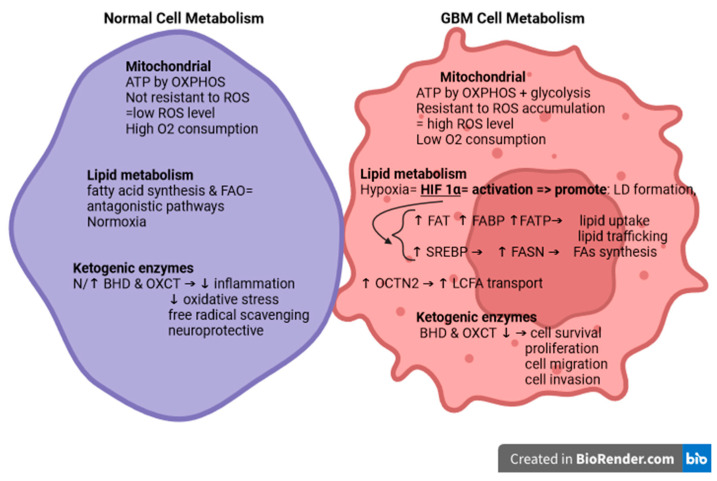
Difference between a normal cell and a GBM cell. In GBM cell mitochondria: ROS levels increase, oxygen consumption is low, and ATP production occurs by glycolysis; FA synthesis and FAO are antagonistic pathways—in GBM both are activated. HIF1 plays a pivotal role in lipid metabolism. It can increase lipid uptake and trafficking, fatty acid synthesis, lipid droplet biogenesis, and lipid signal production, and suppress FAO. Lipid droplet accumulation may be the final result of HIF1 in lipid metabolism. Low expression of ketogenic enzymes in GBM leads to increased survival, proliferation, migration, and invasion of GBM cells. Created in BioRender.

Remodeling of lipid metabolism through changes in FA transport, de novo lipogenesis, storage of LDs, and FAO in order to provide energy is a distinctive sign of cancer, including GBM. Changes in lipid metabolism are associated with different aspects of tumor biology, such as proliferation, migration, and resistance to therapy, and are dependent on the type of tumor or the type of molecular subclass with which it is associated. FAs exert a significant impact on tumors, making disruption of their metabolism a potential strategy for tumor treatment. Consequently, targeting the reprogramming of FA metabolism in tumor cells has become an increasingly prominent focus of research [95,96]. Rapidly proliferating cells require a significant number of FAs to facilitate membrane synthesis and to generate phospholipids crucial for replication. FAs can serve as substrates for mitochondrial ATP synthesis, regulate post-translational modification of lipids, and modulate the function of signaling proteins. Through de novo lipogenesis or exogenous absorption from the surrounding microenvironment, tumor cells obtain FAs. CD36/AG translocase (FAT), plasma membrane AG-binding proteins (FABPpm), and FA transport protein family (FATP)/SLC27 are specialized transporters that facilitate the absorption of FAs from the surrounding microenvironment and are overexpressed in tumors. At the same time, the hypoxia-inducible factor (HIF)-1α promotes FABP expression. Therefore, the tumor microenvironment shows an increased uptake of FAs and, secondarily, an increased number of LDs (Figure 4) [96,97,98,99].

As mentioned above, in the case of normal cells, and in the case of tumor microenvironment cells, LDs have the role of preventing lipotoxicity, thus maintaining lipid homeostasis. At the same time, under conditions of metabolic stress, LDs represent an important source of ATP and NADPH, following the β-oxidation pathways. Acetyl-CoA produced in TCA leads to the generation of NADH and FADH2 for the electron transport chain, with the secondary synthesis of ATP, a quantity six times higher than the oxidation of carbohydrates. Another important source of NADH is the oxidation of citrate diverted from acetyl-CoA under the action of isocitrate dehydrogenase I (IDH1). Thus, sufficient NADH, essential for anabolic metabolism and ROS detoxification, is generated. Thus, hypoxic cells through the overexpression of FABP3 and FABP7 benefit from an increased capture of FAs and a sufficient number of LDs to provide sufficient energy for the recovery of tumor cells, including GBM cells during reoxygenation. At the same time, GBM cells are protected from ROS toxicity by increasing NADPH levels. The in vivo inhibition of FABP3 and FABP7 inhibits the growth of the GBM cell line U87 by reducing the absorption of FAs and, secondarily, by the lack of formation of LDs. This interaction between tumor cells and lipids makes the latter play a crucial role in the preparation of the tumor microenvironment, and, therefore, favors tumor initiation and progression [96,100,101,102].

Analysis of metabolic profiles between low-grade gliomas (LGG) and patient-derived GBM revealed a prevalence of FA catabolism over synthesis in GBM. This observation demonstrates the dual nature of β-oxidation, which encompasses both anabolic and catabolic functions, providing metabolic plasticity to GBM cells, which allows tumor cells to adapt and grow in different microenvironmental conditions [103,104].

Increased de novo lipogenesis, characteristic of tumor metabolism dynamics, leads to overexpression of FA synthetase (FASN), which secondarily promotes the mobility and wound repair abilities of glioma cells. Activation of the phosphoinositol 3-kinase/protein kinase B (PI3K/Akt) signaling pathway reveals positive feedback in maintaining high levels of FASN in GBM cells. It is well known that activation of the PI3K/Akt signaling pathway produces cell proliferation and invasion in malignant gliomas. In orthotopic GBM mouse models, FASN levels were reduced under Temozolomide and Metformin treatment [103,105,106,107,108].

The upregulation of FASN in GBM cells is likely attributable to increased expression of the essential transcriptional regulator sterol regulatory element-binding protein (SREBP) [103,109,110].

SREBP activation plays a crucial role in FA metabolism under hypoxic conditions. In addition, SREBP1 serves as a downstream target of tumor suppressor pathways, including the liver b1/AMP kinase-activated protein kinase (LKB-AMPK) and Akt pathways. AMPK phosphorylates SREBP1, inhibits its activity and, consequently, suppresses tumor growth. AMPK also phosphorylates acetyl-CoA carboxylase (ACC), which leads to the inhibition of FA synthesis. However, in GBM cells, AMPK activation increases ACC activity and levels. Activation of the PI3K/Akt pathway increases the expression of SREBP1 and genes associated with FA synthesis. Furthermore, PI3K hyperactivation and epidermal growth factor receptor (EGFR) mutations promote GBM growth and survival through SREBP-1 activation. These findings suggest that inhibition of SREBP activity could be a promising therapeutic approach [103,111,112].

### 4.1. Carnitine Shuttle System Dysregulation in GBM

As mentioned above, an essential cofactor in lipid metabolic pathways is carnitine. It plays a key role in facilitating LCFA transport across the mitochondrial membrane (Figure 2). Consequently, a deficiency in the carnitine shuttle can lead to a reduced ability of tissues to use LCFA as an energy source. On the other hand, tumor cells grow in lipid-rich microenvironments that give them survival advantages. Therefore, inhibiting the factors involved in the carnitine shuttle could lead to the formation of a microenvironment not favorable to the development of tumor cells and therefore to the slowing down of tumor progression [49,113,114,115].

OCTN2, a membrane transporter of carnitine, is present in both brain cells and GBM cells. The expression of OCTN2 was found to be higher in primary GBM samples from patients, and even more pronounced in samples from patients with recurrent GBM, as compared to the healthy brain [32,116,117].

Fink et al. showed that increased OCTN2 expression in GBM patients correlates with unfavorable outcomes, as demonstrated by decreased tumor cell viability upon OCTN2 silencing by siRNA-mediated activity (preclinical studies using a GBM mouse model). Thus, increased expression of OCTN2 could be a potential prognostic factor for GBM. This in vivo study represents the first demonstration of the antitumor efficacy of the OCTN2/L-carnitine inhibitor Meldonium. Its peculiarity is that no significant side effects have been observed so far. Furthermore, this study suggests the possibility of optimizing GBM therapy through targeted interventions, such as specific inhibition of OCTN2 or drug delivery by targeting OCTN2. It was also shown that L-Carnitine-conjugated nanoparticles promote permeation across the BBB to target glioma cells via OCTN2, resulting in improved antiglioma therapy [118,119].

Bogusiewicz et al. conducted a study aiming to gain a deeper understanding of intermediates in the carnitine transfer system by leveraging data obtained from untargeted lipidomic analyses of brain tumors. Particular attention is paid to factors such as tumor grade (WHO I-II, LGG; WHO III-IV, HGG), the presence of mutations in IDH, and 1p/19q coding. They demonstrated that carnitine levels were significantly increased in HGG compared to LGG, with a ratio of 4.21 in IDH wild-type tumors (IDHwt) compared to IDH mutant tumors (IDHm), with no statistically significant difference between cases with and without the presence of the 1p/19q co-deletion. The mean peak areas for short-, medium-, and long-chain acylcarnitines were greater in HGG compared to LGG, although statistical significance was observed only for short-chain acylcarnitines. Furthermore, their findings showed higher levels of these analytes in IDHwt samples compared to mutants, although the difference was not statistically significant. Carnitine and acylcarnitine levels tended to be higher in tumors with higher malignancy (HGG versus LGG) or in patients with poorer clinical outcomes (IDHwt versus IDHm and with 1p/19q co-deletion versus no co-deletion 1p/19q). The results of this study confirmed that changes in the carnitine transfer system could serve as a crucial factor in measuring the malignancy of gliomas and evaluating clinical prognosis [120].

**Figure 5:**
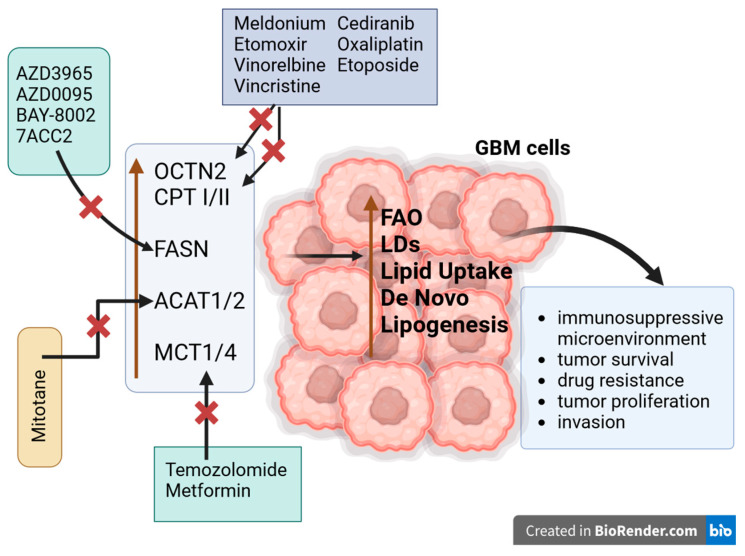
The alterations in FA metabolism in GBM. The metabolic changes in GBM result in increased FAO, LDs, and de novo lipogenesis due to the upregulation of enzymes in their metabolic pathways. Therefore, inhibiting (**X**) these enzymes is expected to enhance survival in patients with GBM. Created in BioRender.

Inhibition of carnitine transport by chemotherapeutic agents such as Vinorelbine and Vincristine resulted in suppression of FAO, which was further potentiated by Etomoxir—a CPT I inhibitor. Consequently, this led to decreased viability and increased apoptosis in glioma cells. Modulation of OCTN2 expression influenced glioma cell survival in an FAO-dependent manner. These results suggest that tumor cell survival is highly dependent on both FAO and OCTN2 activity, indicating that CPT I and OCTN2 could be potential drug targets. Vincristine, Vinorelbine, Cediranib, Verapamil, Oxaliplatin, and Etoposide inhibit OCTN2-dependent carnitine uptake in glioma cells (see Figure 5). Moreover, OCTN2 can transport anticancer drugs, such as Ectoposide or Oxaliplatin [32,118,121,122,123].

While the expression and enzymatic activity of proteins involved in this pathway have been investigated, the specific role and destiny of carnitine and its esters formed during the transport of FAs across the mitochondrial membrane remain poorly understood.

### 4.2. MCTs of KBs in GBM

MCTs are the only known transporters for ketone bodies and are widely distributed throughout the brain. MCT1/2/4 are recognized contributors to cancer development, operate through various mechanisms, and play a critical role in lactate transport. In GBM, maintaining an alkaline intracellular pH is essential to sustain glycolysis. Inhibition of MCT1 and MCT4, which are essential for this pH balance, would effectively prevent glucose metabolism through glycolysis. In vivo studies indicated an upregulation of MCT4 and MCT1 in GBM compared with normal brain parenchyma, oligodendrogliomas, and astrocytomas. Particularly remarkable was the significant increase in MCT4 levels observed in necrotic tissues of GBM tumors. These findings suggest the potential for strategies targeting MCT4 and MCT1 to provide new avenues for the development of new therapeutic targets. MCT1 inhibitors, including AZD3965, BAY-8002, and 7ACC2, bind to distinct conformations of MCT1, either outward or inward (Figure 5). However, all three inhibitors directly occupy the substrate binding site. Goldberg et al. showed that AZD0095 exhibits outstanding potency, high selectivity for MCT1, favorable secondary pharmacology, a well-defined mechanism of action, suitable properties for oral administration in clinical settings, and promising preclinical efficacy when used in combination with cediranib [124,125,126,127,128].

Current research on the role of MCTs in GBM is limited; however, the many implications associated with their function underscore the need for further investigation. Gaining a deeper understanding of MCTs in GBM is crucial for the development of new inhibitors. While there are several inhibitors targeting MCTs, there is currently a lack of research into potential therapeutic drugs specifically tailored for GBM.

### 4.3. The Role of Ketogenic Enzymes in Glioblastoma

Given the dynamic and nutrient status-sensitive nature of ketone metabolism, there is considerable interest in exploring its biological connections to cancer. This interest stems from the potential for precision-guided nutritional therapies in cancer treatment [82].

Ketogenic enzymes, in turn, contribute to cell maintenance and energy supply, in order to prepare the microenvironment necessary for the development of GBM cells. This, together with the Warburg effect, underscores the importance of ketogenic metabolism in driving GBM progression. [52,93,129].

To obtain a clearer understanding of ketogenic enzymes in human gliomas and especially in GBM, in Table 2 we investigate the expression levels of several key enzymes involved in this metabolic pathway.

## 5. Conclusions

The metabolic shifts observed in tumor cells are significant, primarily dictated by genetic factors. Our therapeutic aim revolves around altering these genetic determinants to enhance the cells’ susceptibility to anticancer treatments.

The remodeling of lipid metabolism in GBM involves changes in FAO, FA transport, de novo lipogenesis, and LD storage. By increasing the expression of FA transporters and the transporters of the molecules involved in their supply, there is an increase in the content of FAs in the tumor microenvironment. The excess of FAs is stored in the form of LDs, which, in turn, will be split to generate energy. Thus, the hyperexpression of HIF-1α in GBM leads to the hyperexpression of FAT, FABPpm, FATP, and, secondarily, the increase in the absorption of FAs and the formation of LDs in the tumor microenvironment. LDs prevent lipotoxicity and provide a source of ATP and NADPH in conditions of metabolic stress. The inhibition of FABP3 and FABP7 leads to the reduction of FA absorption and inhibits the growth of GBM cells.

Analysis of metabolic profiles between LGG and GBM reveals an increase in FA catabolism in GBM, providing metabolic plasticity for tumor adaptation and growth in different microenvironments. Through the PI3K/Akt signaling pathway, there is an overexpression of FASN with the promotion of de novo lipogenesis, which is associated with increased GBM cell invasion. At the same time, there is an increase in the expression of OCTN2, an important transporter of carnitine. Inhibition of OCTN2 has demonstrated antitumor efficacy in preclinical studies and could be a potential prognostic factor and therapeutic target for the treatment of GBM.

The role of KBs and ketogenesis enzymes in GBM metabolism is equally important, but still poorly understood, so new studies are needed regarding their expression and functions in GBM, as well as the potential of ketogenic therapies, such as ketogenic diets for this pathology. MCTs serve as key transporters for KBs across the BBB. Targeting MCTs, particularly MCT2 and MCT4, presents a novel approach in managing GBM cell metabolism.

The ongoing discovery of new therapeutic targets aimed at altering tumor metabolism underscores the importance of ongoing research in this area. In recent years, attention has been paid to metabolic reprogramming in GBM, as it has become increasingly evident that it plays a substantial role in the pathogenesis of these aggressive tumors. Investigations into the use of lipid metabolism for GBM have been limited, largely due to inadequate understanding of the physiological functions of lipids and lipid-related pathways in the brain and GBM. Therefore, future investigations focusing on therapeutic strategies targeting lipid metabolism pathways may provide new and practical concepts for GBM therapy.

## Figures and Tables

**Table 1:** Molecular aspects of the MCT family of transporters in the brain and their main role.

Gene Name	Protein Name	Main Substrates	Main Role
SLC16A1	MCT1	Lactate, ketone bodies, and pyruvate	– Predominant expression in endothelial cells with a barrier role
SLC16A2	MCT8	T2, rT3, T3, T4	– High affinity to transport thyroid hormones = specific thyroid hormone transporters
SLC16A3	MCT4	Ketone bodies, lactate, and pyruvate	– Predominant expression presence in astrocytes
SLC16A4	MCT5	-	– Expression is elevated in certain GBM cells
SLC16A6	MCT7	Ketone bodies	– Transporter of ketone bodies
SLC16A7	MCT2	Lactate, ketone bodies, and pyruvate	– Predominant expression in neurons – High susceptibility to hypoxia – High sensitivity to intracellular pH
SLC16A9	MCT9	Carnitine	– Act a carnitine efflux transporter
SLC16A14	MCT14	-	– Hypothesis: MCT14 is a neuronal aromatic amino acid transporter
Derived from references [48,83,85,86,87,88,89]			

**Table 2:** The ketogenic enzymes and their role in cancer and in GBM.

References	Aim	Analyzed Data	Suggested Mechanism of Findings	Significance and Future Research
**ACAT1 & ACAT2**				
Wang et al. [129]	– ACAT1 expression and myeloid cell ratio	ACAT1 ITGAM * CXCL1 * MDSCs *	↑ ACAT1 expression → ↑ myeloid cell ratio ↑ myeloid marker ITGAM → ↑ myeloid cell infiltration in tumor tissue CXCL1 = crucial role in the induction of MDSCs and accelerated tumor growth	– Manipulating MDSCs has emerged as a promising target for the development of anticancer therapies – ACAT1 may play a potential role in the immune microenvironment – CXCL1 contributes to the development of an immunosuppressive microenvironment and facilitates tumor progression. It is a potential prognostic biomarker and therapeutic target for patients with GBM
Kou et al. [39]	– Suppression of ACAT and suppression of SREBP-1 = blocked GBM growth	SREBP-1 LDs ACAT	LDs ↑ in GBM patient tissues (infrequently in LGG, and undetectable in normal brain tissues) – The formation of LDs is a signature feature of GBM – ↑ LDs = poor survival – ACAT1 protein level is correlation with LD formation – ACAT2 = no expression in GBM patient tumor tissues/rarely expressed – SREBP-1 = highly activated in GBM	– SREBP-1 = a potential therapeutic target in malignancies – Inhibiting ACAT1 to block cholesterol esterification represents a promising therapeutic strategy for treating GBM by suppressing SREBP-1 – LDs could potentially be mobilized when cancer cells encounter a harsh microenvironment
Ohmoto et al. [130]	– Roles of ACAT1 in human GBM cell line U251-MG – Role of K604	ACAT1 K604 AKT ERK1/2 *	– ACAT1 = expressed in human GBM tissues – At low cell density, proliferation of GBM cell line U251-MG is significantly inhibited by K604 treatment – At medium and high cell densities, K604 had no effect on the proliferation of GBM cell line U251-MG – Phosphorylation of AKT and ERK1/2 = inhibited by K604 in a dose-dependent manner – The activation of AKT and ERK1/2 may be associated with refractory GBM	– ACAT1 may be a promising therapeutic target for GBM – Further research is needed to elucidate the molecular mechanisms underlying the inhibition of ERK1/2 and AKT phosphorylation by K604
Löhr et al. [131]	– ACAT1 and the GBM microenvironment	ACAT1 GBM IDHw GBM IDHm-R132H SREBP-1 LD CD68	– ACAT1 = more pronounced in microglia and macrophages rather than in tumoral cells – Expression of ACAT1 ↑ in GBM compared to LGG – No expression of ACAT1 in normal brain tissues – ↑ LD in GBM – IDHm status exhibited a near absence of LD accumulation, whereas GBM showed an abundance of LDs	– The tumor microenvironment, including the macrophage/microglia may serve as a therapeutic target – Mitotane could be particularly promising for GBM patients who have exhausted other treatment options – The in vitro impact of ACAT1 inhibition on macrophage polarization holds significant interest
Bemlih et al. [67]	– Expression of ACAT1 in U87, A172 and GL261 glioma cell lines and in normal human astrocytes	ACAT1 CD80 CD86 MHC * class I	– ACAT1 expression is inhibited by Avasimibe in glioma cell lines – ACAT1 expression was unchanged in normal human astrocytes – Avasimibe = ↑ the expression of costimulatory molecules (CD80, CD86), and ↑ MHC class I (characteristic of immunogenicity of glioma cells)	– The effect of Avasimibe on glioma is dose-dependent (was nearly abolished at 7.5 μM) – Inhibition of ACAT1 = ↑ immunogenicity of tumor cells. This finding suggests that such inhibition could be valuable in enhancing T-cell responses to tumors in vivo – Targeting cholesterol metabolism through the inhibition of ACAT-1 activity in GBM patients presents a novel opportunity for controlling glioma progression
**HMG CoA Synthase & HMG CoA Lyase**				
Zhao et al. [64]	– Role of HMG CoA Synthase and HMG CoA lyase in cancer	HMG CoA Synthase BRAF^V600E^	– HMG CoA Synthase shows a positive correlation with tumoral cell growth – The dehydroacetic acid selectively inhibits the proliferation and tumor growth of cells expressing BRAF^V600E^	– HMG CoA Synthase = play a oncogenic role – Ketogenic HMG CoA Synthase-HMG CoA Lyase-acetoacetate axis is a promising therapeutic target in treatment of BRAF^V600E^ positive human cancers
Zhou et al. [132]	HMG CoA Syn-thase 1 in cancer	HMG CoA Synthase 1 Fibroblast CD8+	– HMG CoA Synthase = highly expressed and negatively correlated with the prognosis in cancer – Compared to adjacent control tissues, HMG CoA Synthase 1 was underexpressed in GBM tissues – The infiltration levels of CD8+ T cell and cancer associated fibroblast = closely associated with HMG CoA Synthase 1 expression	– High HMG CoA Synthase 1 expression could reduce the sensitivity to drugs in cancer – HMG CoA Synthase 1 = had impacts on cell proliferation and immunity
**BHD & OXCT1**				
Chang et al. [66]	– Expression of BHD and OXCT in glioma WHO grade III and in GBM	BHDOXCT GFAP	↓ expression of BHD in GBM ↓ expression of OXCT in GBM	– Patients with low expression of BHD and OXCT in gliomas may respond better to adjuvant therapy, such as a ketogenic diet → further investigations utilizing animal models and/or conducting large-scale clinical trials are essential to validating these findings
Schwartz et al. [133]	– Expression of BHD and OXCT in GBM patients and a Ketogenic diet	BHDOXCT	– Patient 1 (original tumor) = ↓ expression of OXCT and BHD in GBM, but still exhibits positive expression – Patient 2 = The majority of tumoral cells are positive for OXCT and BHD → some GBM cells could metabolize ketone, thus obtaining energy for their continued growth	Ketocal = ↓ the blood glucose and ↑ blood ketones, initially ↓ in weight (6%), but after then the patients’ weight was stabilized → use an adjunctive therapy for GBM shows promise. The question arises whether GBM cells can utilize ketones for proliferation and growth?

* ITGAM = integrin subunit alpha M; CXCL-1 = C-X-C motif chemokine ligand 1; MDSCs = myeloid-derived suppressor cells; ERK1/2 = extracellular signal regulated kinase ½; MHC = major histocompatibility complex; ↑ = increase; ↓ = decrease; → = results.
